# Interplay of LIS1 and MeCP2: Interactions and Implications With the Neurodevelopmental Disorders Lissencephaly and Rett Syndrome

**DOI:** 10.3389/fncel.2019.00370

**Published:** 2019-08-14

**Authors:** Liraz Keidar, Gabi Gerlitz, Aditya Kshirsagar, Michael Tsoory, Tsviya Olender, Xing Wang, Ying Yang, Yu-Sheng Chen, Yun-Gui Yang, Irina Voineagu, Orly Reiner

**Affiliations:** ^1^Department of Molecular Genetics, Weizmann Institute of Science, Rehovot, Israel; ^2^Department of Veterinary Resources, Weizmann Institute of Science, Rehovot, Israel; ^3^Beijing Institute of Genomics, Chinese Academy of Sciences, Beijing, China; ^4^School of Biotechnology and Biomolecular Sciences, The University of New South Wales, Sydney, NSW, Australia

**Keywords:** lissencephaly, Rett syndrome, LIS1, MeCP2, genetic interactions

## Abstract

*LIS1* is the main causative gene for lissencephaly, while *MeCP2* is the main causative gene for Rett syndrome, both of which are neurodevelopmental diseases. Here we report nuclear functions for LIS1 and identify previously unrecognized physical and genetic interactions between the products of these two genes in the cell nucleus, that has implications on MeCP2 organization, neuronal gene expression and mouse behavior. Reduced LIS1 levels affect the association of MeCP2 with chromatin. Transcriptome analysis of primary cortical neurons derived from wild type, *Lis1*±, *MeCP2−/y*, or double mutants mice revealed a large overlap in the differentially expressed (DE) genes between the various mutants. Overall, our findings provide insights on molecular mechanisms involved in the neurodevelopmental disorders lissencephaly and Rett syndrome caused by dysfunction of LIS1 and MeCP2, respectively.

## Introduction

A continued challenge in the post genome era is to understand how gene expression and protein interactions sculpt behavior, which is of prime interest in case of brain diseases. Here we investigated a novel physical and functional interaction between the protein products of two neurodevelopmental disease associated genes *LIS1* and *MeCP2.*

*LIS1* (*Lissencephaly 1*) (officially known as *PAFAH1B1)* was the first gene to be identified as involved in a neuronal migration disorder (Reiner et al., 1993) (review Reiner, 2013). The lissencephaly pachygyria spectrum of diseases defines a variety of brain malformations that cause relative smoothness of the brain surface. These are relatively rare brain malformations, resulting in severe developmental delays, and most patients do not reach measurable developmental stages. Deletions of one allele of *LIS1* result in lissencephaly in humans (Reiner et al., 1993), and impair neuronal migration in mice (Hirotsune et al., 1998; Cahana et al., 2001). There is dosage sensitivity at the *LIS1* locus; increased gene dosage also damages proper brain development both in humans and in mouse (Bi et al., 2009). Point mutations in *LIS1* are relatively rare, and may result in less severe consequences than gene deletions (Sapir et al., 1999; Saillour et al., 2009; Philbert et al., 2017). *Lis1−/*+ mice display cortical, hippocampal and olfactory bulb disorganization resulting from delayed neuronal migration (Hirotsune et al., 1998). These mice are fully viable, breed and have no motor impairment (Paylor et al., 1999). *Lis1*± filopodia on immature hippocampal neurons *in vitro* exhibited reduced density, length and RhoA dependent impaired dynamics compared to wild-type (Sudarov et al., 2013). Deletion of LIS1 in adult mice results in lethality (Hines et al., 2018), whereas a postnatal deletion specific to neurons affects the structure and cellular composition of the hippocampus (Sudarov et al., 2018).

Mutations in the X-linked *MeCP2* (methyl-CpG-binding protein 2) gene result in Rett syndrome, a relatively common progressive neurodevelopmental disorder (Amir et al., 1999). Most of the mutations occur *de novo*, and missense mutations that cause Rett syndrome are concentrated in two discrete clusters coinciding with interaction sites for partner macromolecules: the methyl-CpG binding domain (MBD), involved in DNA binding (Nan et al., 1993) and the NCoR/SMRT interaction domain (Lyst et al., 2013; Tillotson et al., 2017). The majority of the patients are female, suggesting early lethality for mutated *MeCP2* male embryos (Chahrour and Zoghbi, 2007). Interestingly, similar to *LIS1* mutations, the *MeCP2* locus is also dosage sensitive. Gains in MeCP2 dosage results in neurological disorders that are clinically similar to loss in MeCP2 (Chahrour and Zoghbi, 2007). The disease involves normal embryonic and early postnatal development followed by development stagnation during the second year of life (Chahrour and Zoghbi, 2007).

MeCP2 (methyl-CpG-binding protein 2) was initially identified as a protein that binds methylated DNA sites (Lewis et al., 1992), yet its mode of action, which has proven to be cell type specific, has not been easy to decipher. MeCP2 is an abundant protein in neuronal nuclei, which is as plentiful as histone octamers (Skene et al., 2010). MeCP2 binding induces chromatin compaction that is modulated in a cell specific manner that is strikingly similar to the binding of histone H1 to chromatin (Georgel et al., 2003; Brero et al., 2005; Ghosh et al., 2010; Linhoff et al., 2015). The binding of MeCP2 to chromatin is altered by mutations causing Rett syndrome (Marchi et al., 2007; Nikitina et al., 2007; Kumar et al., 2008). During development, *MeCP2* mutant mice exhibit increased levels of histone H1 that may partially compensate for the loss in MeCP2, and as such explain for delay in major disease phenotypes to the postnatal stage (Skene et al., 2010). Recent evidence indicated that MeCP2 can not only induce chromatin compaction, but that targeted binding of MeCP2 to specific loci may elicit extensive chromatin unfolding (Brink et al., 2013). In addition, MeCP2 known protein interactors connect it to repression or to activation of transcription (Nan et al., 1997; Jones et al., 1998; Chahrour et al., 2008; Kim et al., 2013; Lyst et al., 2013; Maxwell et al., 2013; Lyst and Bird, 2015; Mahgoub et al., 2016; Ludwig et al., 2017; Rajavelu et al., 2018), RNA splicing (Jeffery and Nakielny, 2004; Maunakea et al., 2013; Li et al., 2016; Cheng et al., 2017; Wong et al., 2017; Zheng et al., 2017), as well as microRNA binding (Khan et al., 2017).

LIS1 was found to be a non-catalytic subunit of platelet-activating factor acetyl-hydrolase (PAFAH1B1) (Hattori et al., 1994), as well as a tubulin interacting protein that modulates the dynamics of microtubules (Sapir et al., 1997). Most of the known functions of the LIS1 protein are cytoplasmic and relate to its interactions with the molecular motor cytoplasmic dynein and its modulators (Willins et al., 1997; Efimov and Morris, 2000; Faulkner et al., 2000; Niethammer et al., 2000; Ahn and Morris, 2001; Han et al., 2001; Hoffmann et al., 2001; Coquelle et al., 2002; Tarricone et al., 2004; Grabham et al., 2007; Yamada et al., 2008; McKenney et al., 2010; Shmueli et al., 2010; Yang et al., 2010; Murdoch et al., 2011; Pandey and Smith, 2011; Egan et al., 2012; Huang et al., 2012; Rompolas et al., 2012; Splinter et al., 2012; Trokter and Surrey, 2012; Reiner and Sapir, 2013; Kuijpers et al., 2016; DeSantis et al., 2017; Infante et al., 2018).

Here we show that LIS1, in addition to its cytoplasmic localization, exhibits a nuclear localization and demonstrated that it can interact with histone H1. Lack of MeCP2 protein causes an upregulation of histone H1, suggesting a possible compensatory mechanism between these two proteins, therefore, we postulated that LIS1 could also interact with MeCP2. Indeed, we demonstrate that the two proteins physically interact and that LIS1 can affect MeCP2 binding to chromatin and transcriptional regulation in primary neuronal cultures as well as MECP2-dependent behavioral changes in behaving animals using conditional deletion adult single and double mutant mice (*MeCP2−/y, Lis1*±, and double). Overall, our studies uncover a novel functional interaction between LIS1 and MECP2. Overall, our novel findings promote the understanding of the molecular mechanisms involved in the neurodevelopmental disorders lissencephaly and Rett syndrome caused by dysfunction of LIS1 and MeCP2, respectively.

## Results

### LIS1 Interacts With Histone H1 and MeCP2

**FIGURE 1:**
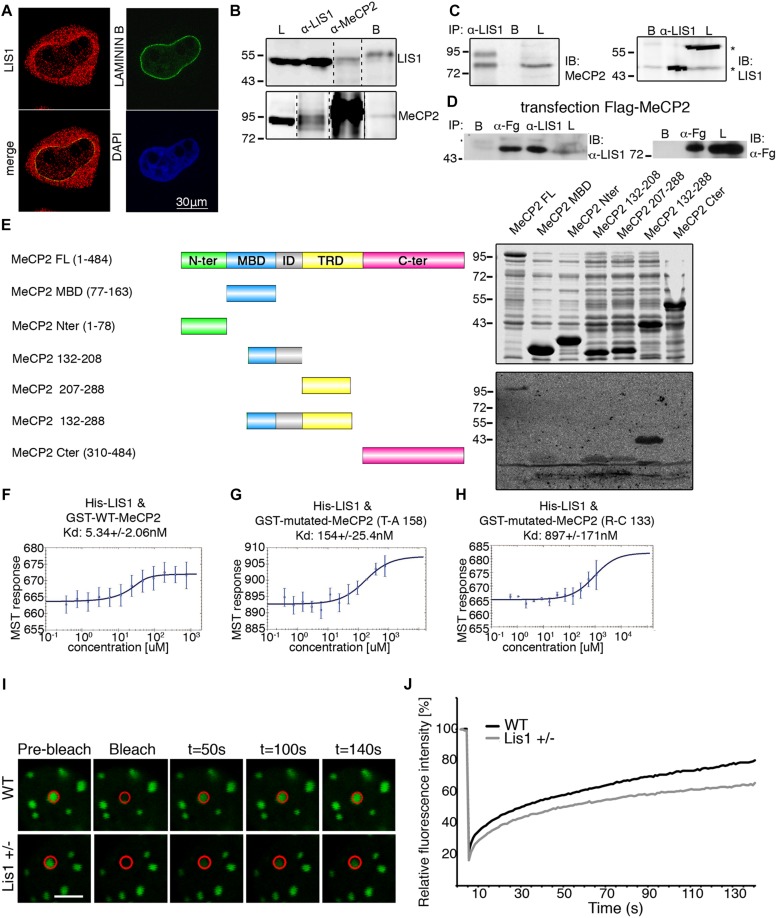
LIS1 and MeCP2 interaction. **(A)** Nuclear localization of LIS1. HeLa cells were fixed and immunostained for LIS1 (Ab210, red) and Lamin B (green). DNA was stained with DAPI (blue). **(B)** Co-IP of LIS1 and MeCP2 from cross-linked P1 brains lysates. P1 mouse brains were fixed in 1% PFA, sonicated and immunoprecipitated using anti-LIS1 or anti-MeCP2 antibodies. Western blot analysis of the lysates (L), respective immunoprecipitations, and control beads (B) is presented. **(C)** Anti-LIS1 antibodies immunoprecipitated endogenous MeCP2 from mouse brain nuclei. **(D)** Anti-FLAG (Fg) antibodies immunoprecipitated flag-tagged MeCP2 from transfected HEK293 cells and co-immunoprecipitated endogenous LIS1. **(E)** LIS1 interacts directly with MeCP2 in a defined tandem repeat domain *in vitro*. Recombinant full-length (FL) or indicated fragments of MeCP2 were expressed in bacteria. Bacterial lysates were separated by SDS-PAGE, the gels were either Coomassie-blue stained (top box) or subject to Far-western analysis (bottom box). MeCP2 fragments (132–208, 207–288, and 132–288) bind to LIS1, note the stronger signal with the fragment containing the tandem repeat (132–288). **(F,G)** Microscale Thermophoresis (MST) interaction analyses of LIS1 with MECP2. Fluorescently labeled His-LIS1 was incubated with increasing concentrations of recombinant MeCP2, wild-type **(F)**, T158A **(G)**, R133C **(H)**. The MST response was used to calculate the indicated dissociation constants (Kd). **(I,J)** FRAP analyses of MeCP2-GFP in WT or *Lis1*± cortical neurons. **(I)** Representative images of bleached cells in which the bleached region is marked by a red circle. **(J)** Relative average fluorescence intensity over time (*n* = 24), Ctl black line, *Lis1* shRNA gray line. The scale bar is 5 mm.

The LIS1 protein has been usually discussed in the context of its cytoplasmic activities (Willins et al., 1997; Efimov and Morris, 2000; Faulkner et al., 2000; Niethammer et al., 2000; Ahn and Morris, 2001; Han et al., 2001; Hoffmann et al., 2001; Coquelle et al., 2002; Tarricone et al., 2004; Grabham et al., 2007; Yamada et al., 2008; McKenney et al., 2010; Shmueli et al., 2010; Yang et al., 2010; Murdoch et al., 2011; Pandey and Smith, 2011; Egan et al., 2012; Huang et al., 2012; Rompolas et al., 2012; Splinter et al., 2012; Trokter and Surrey, 2012; Reiner and Sapir, 2013; Kuijpers et al., 2016; DeSantis et al., 2017; Infante et al., 2018). Yet, a significant portion of the protein can be detected in the nucleus (Figure 1A). Biochemical fractionations demonstrate that LIS1 can be detected both in the cytoplasm and in the nucleus (Supplementary Figure 1). Nuclear and cytoplasmic fractions of mouse embryonic stem cells were blotted with anti-LIS1 antibodies, anti-nanog antibodies (a nuclear transcription factor), and anti-GAPDH. Further biochemical subfractionations in N2a and HeLa cells demonstrate that LIS1 is detected both in the nucleoplasm and in the nuclear envelope using as controls antibodies detecting Lamin B, Nup62, and Lap2b (Supplementary Figure 2).

Far-western analysis revealed specific interactions between LIS1 and nuclear envelope proteins (Supplementary Figure 3). Far-western analysis coupled with DEAE-fractionation and mass-spectrometry analysis revealed that LIS1 interacts with histone H1 subtype H1E (Supplementary Figures 4, 5). Based on these findings, we postulated that LIS1 may also interact with MeCP2. LIS1 and MeCP2 were co-immunoprecipitated following cross-linking using either anti-LIS1 antibodies or anti-MeCP2 antibodies from mouse brain lysates (Figure 1B and Supplementary Figure 6). The size of the endogenous MeCP2 cross-reactive band was approximately 85 kDa, similar to that originally reported (Lewis et al., 1992). In addition, anti-LIS1 antibodies immunoprecipitated MeCP2 from brain nuclear extracts (Figure 1C). In the lysates we noted two sizes of LIS1 cross-reactive bands, one of 46 kDa and the second of 56 kDa (Figure 1C marked with asterisk). We could not demonstrate a reciprocal immunoprecipitation using anti-MeCP2 antibodies, therefore, we transfected HEK293 cells with FLAG-tagged MeCP2, which successfully immunoprecipitated endogenous LIS1 (Figure 1D). Next, we used a series of bacterially expressed recombinant MeCP2 proteins to map the interaction domain using far-western analysis (Figure 1E and Supplementary Figure 7). The initial survey revealed that two regions may be involved in LIS1 binding, the first region included the methyl binding domain (MBD) and the intervening domain (ID) and the second region included the transcription repression domain (TRD). Alignment of the amino acid sequence of the two regions indicated some similarity in the sequence of 55 amino acids (Supplementary Figure 8). Therefore, we tested the possibility that whereas LIS1 can interact with either of these regions, the interaction with the tandem repeat may increase the binding (Figure 1E). The minimal fragment that included all these tandem repeats extended between amino acids 132–288 and its interaction with ^35^S- labeled LIS1 resulted in a strong signal (Figure 1D). The direct physical interaction between LIS1 and MeCP2 was verified by analyzing the respective purified recombinant proteins by MicroScale Thermophoresis (MST). The interaction between LIS1 and MeCP2 was of high affinity (5.34 ± 2.06 nM) (Figure 1F). Two different pathogenic mutations of the MeCP2 protein (MeCP2 T158A, Goffin et al., 2011 and MeCP2 R133C, Ballestar et al., 2000), with amino acid substitutions that are located in the above designated LIS1-binding domain, exhibited a decreased interaction with LIS1. The interaction affinities of MeCP2 mutated in amino acid 150 or 133 were reduced by an order of magnitude (Figures 1G,H).

In view of the interaction of LIS1 with either histone H1 or with MeCP2, we considered that one possible functional outcome for the LIS1-MeCP2 interaction would be to affect the association of MeCP2 with chromatin. To address this issue, we used fluorescence recovery after photobleaching (FRAP) to study the dynamics of MECP2 binding using wild type primary neurons in comparison with cells with reduced levels of LIS1 (*Lis1*±). As previously demonstrated, MeCP2-GFP fusion proteins have a prominent chromocenter localization which is suitable for quantitative analysis of their DNA binding properties (Klose et al., 2005; Marchi et al., 2007; Kumar et al., 2008; Becker et al., 2016; Sheikh et al., 2016). Neurons with half LIS1 dosage exhibited a slower recovery rate of the photobleached MeCP2-GFP signal than control cells with *t50* (time required for the recovery of 50% of the initial fluorescent signal) of 65.46 ± 9.2 and 33.2 ± 3.9 s, respectively (Student’s *t*-test, *p* ≤ 0.005) (Figures 1I,J). Our studies indicate that LIS1 affects the binding of MeCP2 to chromatin.

### LIS1 and MeCP2 Affect Gene Expression and Share Common Target Genes

As detailed above, LIS1 affects the interaction of MeCP2 with chromatin. Since MeCP2 is known to affect gene expression (Nan et al., 1997; Chahrour et al., 2008), we decided to compare the effects of reduced levels of LIS1, MeCP2 and the double mutants on the cellular transcriptome. We carried out this analysis in primary neurons from *Lis1*±*, MeCP2−/y*, and double mutant mice for the following reasons: (1) Knocking-down LIS1 in primary neurons affected MECP2 chromatin binding as measured by FRAP analysis (Figure 1). (2) Primary neurons comprise a relatively more uniform cellular composition compared to whole brains. (3) *Lis1*± mice have a well-established neuronal developmental phenotype (Hirotsune et al., 1998; Cahana et al., 2001; Gambello et al., 2003; Kholmanskikh et al., 2006; Valdes-Sanchez et al., 2007; Yamada et al., 2008; Youn et al., 2009; Gopal et al., 2010; Sudarov et al., 2013) and (4) Although not well-established, there are some evidence from the literature that there may be deficits in the developmental process of MeCP2 mutant neurons (Matarazzo et al., 2004; Squillaro et al., 2012; Mellios et al., 2018a, b; Sharma et al., 2018). RNA was extracted from primary cortical neurons derived from wildtype, *Lis1*±*, MeCP2-/y*, and double mutant mice.

**FIGURE 2:**
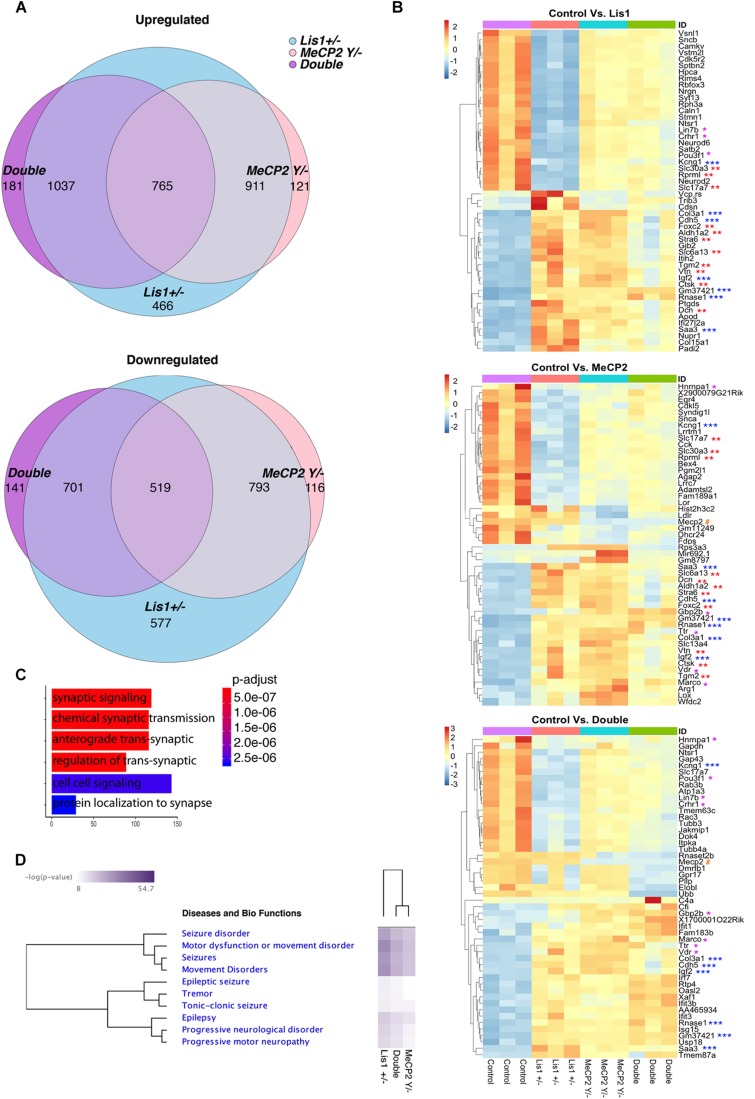
An integrated RNA sequencing approach to identify LIS1-MeCP2 genetic interaction in mouse primary neurons. **(A)** The number in each circle of 3-set Venn diagrams represents the differentially expressed (DE) genes between the different comparisons (Control versus *Lis1*±, *MeCP2 Y/–* and Double). For Up and Down regulated genes with Deseq2 *p*-adjusted value < 0.05 and log2 foldchange more than 1 and less than –1 were determined, respectively. The overlapping numbers stand for the mutual differentially expressed genes between the different comparisons and non-overlapping numbers belong to the specific genes unique to each condition. **(B)** Hierarchical clustering and heatmap representation of top 50 differentially expressed genes in the primary neurons of the indicated groups (Control vs. *Lis1*±, Control vs. *MeCP2 Y/–* and Control vs. Double). The color-coded scale represents the *z*-score of DESeq2 log normalized expression values. The top differentially expressed common genes between Control vs. Double and Control vs. *Lis1*± or *MeCP2 Y/–* are marked with asterisk (^*^), Control vs. *Lis1*± and Control vs. *MeCP2 Y/–* are marked with (^∗∗^) and common differentially expressed genes across all the comparisons are marked with (^∗∗∗^). MeCP2 is denoted with #. **(C)** Enriched GO terms for top common significantly upregulated genes in Control group, in comparison to all the mutants with the range of associated *p*-adjusted values. **(D)** Ingenuity pathway analysis of the selected enriched terms in the Diseases and bio-functions category with the hierarchical clustering and representative heatmap of *p*-values in all the mutant groups.

Overall, we noted that the number of significantly differentially expressed (DE) genes relative to WT neurons was the highest in neurons with reduced LIS1 levels (a total of 5799 genes) (Figure 2A and Supplementary Figure 7). The vast majority of genes DE in the *MeCP2 y/−* neurons were shared with genes DE in *Lis1*± neurons (95%, *p* = 0, hypergeometric test), as well as the genes DE in double mutant neurons (93.5%, *p* = 0, hypergeometric test). Notably, the trend of gene expression changes was preserved between the three genotypes. Genes upregulated in *MeCP2 y/−* neurons significantly overlapped genes upregulated in *Lis1*± neurons (91.7%, *p* = 0, hypergeometric test) as well as genes upregulated in double mutant neurons in comparison with *Lis1*± (89.5%, *p* = 0, hypergeometric test). Conversely, genes downregulated in *MeCP2 y/−* neurons significantly overlapped genes downregulated in *Lis1*± neurons (91.8%, *p* = 0, hypergeometric test) as well as in double mutant vs. *Lis1*± comparison (89.6%, *p* = 0, hypergeometric test).

Among the top 50 DE genes of the three groups (Figure 2B and Supplementary Figure 9), several were significantly dysregulated in all three comparisons (i.e., each mutant vs. wild type), while many others were significant in two comparisons (Figure 2B). Pathway analysis of the altered genes identified that the most significant GO terms are related to synapses and included synaptic signaling as well as cell-cell signaling (Figure 2C). Ingenuity © analysis revealed a strong association of DE genes with neuronal diseases such as seizures, motor dysfunction and epilepsy (Figure 2D), all are relevant to the phenotypes associated with diseases causes by *Lis1* and *MeCP2.* This global trend was also observed for genes DE in *MeCP2−/y* neurons and was preserved in the double mutants (Figure 2D). In summary, our findings indicate that changes in LIS1 and MeCP2 dosage in primary neurons, modulate the expression of an overlapping groups of genes associated with the pathologies that are shared between the two diseases.

### LIS1 and MeCP2 Affect Behaviors in Adulthood

Most of the published studies have been focused on the function of MeCP2 in adulthood (McGraw et al., 2011; Nguyen et al., 2012; Robinson et al., 2012), since the human and mouse phenotypes including abnormal coordination, repetitive movements, as well as progressive microcephaly, are observed at the postnatal stage. Heterozygous *Lis1* mice did not differ from control animals in most of the reported studies (Sudarov et al., 2018), yet some studies did report some effects; for example, heterozygous *Lis1* mice exhibited deficits in dendritic protrusion density and in some social interactions (Sudarov et al., 2013); whole body inactivation of *Lis1* in adulthood resulted in an acute neurological disorder and rapid death (Hines et al., 2018) (and our unpublished results). To evaluate the interactions between MeCP2 and LIS1 on behaviors in adulthood we conducted a series of tests using a conditional whole-body approach (ubiquitin-CreERT2) with *Lis1fl/*+*, MeCP2fl/y*, double mutants, and control mice (resulting after inducing the deletion in *Lis1−/*+*, MeCP2−/y* and double mutant mice). We confirmed that the conditional deletion occurred by DNA analysis and measuring a reduction in LIS1 mRNA levels (Supplementary Figures 10A,B). Despite a complete *Lis1−/−* tamoxifen -induced deletion, we observed relatively high levels of the LIS1 protein that were retained in the primary neurons treated with tamoxifen. LIS1 was similarly present in extracts from the behaving *Lis1*± mice (Supplementary Figures 10C,D, respectively), as well as residual levels of MeCP2 (Supplementary Figure 10D). The mice were subjected to behavioral tests 1 month after the tamoxifen treatment.

**FIGURE 3:**
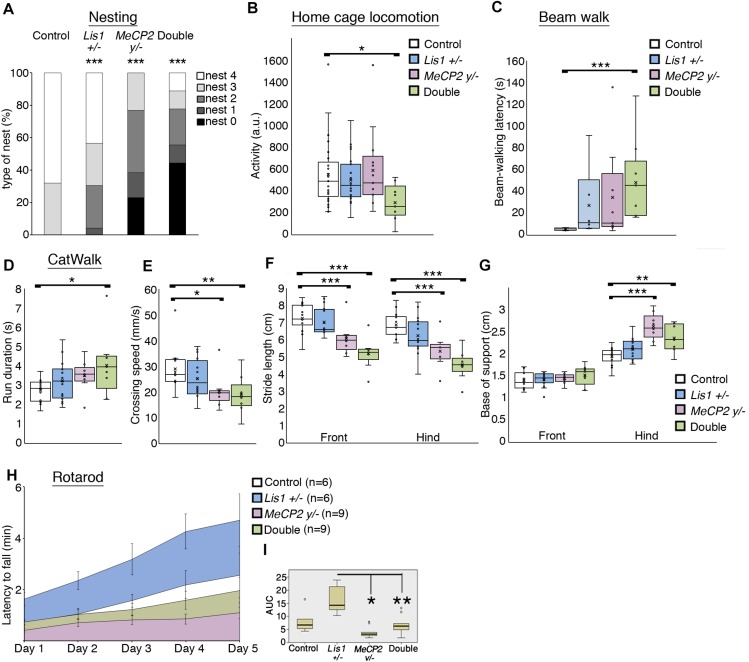
LIS1-MeCP2 interaction, implications on adult brain. **(A)** The degree of nest building was assessed on a scale of 0 – 4 (Deacon, 2006). Nesting ability is presented in the distribution graph. Both the single and the double mutants exhibited impaired nesting ability. **(B)** The mean locomotion activity during the dark (active) diurnal phase is presented. The double mutant mice were significantly less active than the WT mice during the dark phase. **(C)** The mean latency to cross the beam in the balanced beam test is presented. The double mutant mice exhibited a significant higher latency to cross over. **(D–G)** CatWalk parameters are presented. **(D)** The mean run duration, double mutants differed from WT. **(E)** Mean speed, the double mutant and the *MeCP2 cKO* mice were significantly slower than the WT. **(F)** The stride length of front paws (on left) and the hind paws (on right) are presented. Stride length of both hind and front paws of the *MeCP2 cKO* and the double mutant was shorter than that of WT mice. **(G)** The base of support (BOS) of front paws (left) and the hind paws (right) are presented, the hind paws BOS of the *MeCP2 cKO* and the double mutant mice was higher as compared to WT. **(H,I)** The latency to fall in the rotarod test is presented. **(H)** The latency to fall over different days is presented. **(I)** Comparison between the different genotypes. Heterozygotes *Lis1* exhibited significant increased latency to fall as compared with *MeCP2 cKO* and double mutants mice. Data are shown in **(B–G)**, and **(I)** as Box plots [box = 25, 50 (median), 75 percentiles; whiskers = 5–95 percentiles] and **(H)** as mean ± SEM. ^*^p < 0.05, ^∗∗^*p* < 0.01, ^∗∗∗^*p* < 0.001, as compared with controls.

Nesting is an individual goal-directed behavior found in mice, this behavior was impaired in conditional *MeCP2−/y* (McGraw et al., 2011). Our data indicate that all the mutant mice exhibited significantly poor nest building than controls (Figure 3A). *Lis1^–/+^* were somewhat less affected, *MeCP2^–/y^* were more affected and the double mutant mice were the most impaired in this task [Figure 3A, WT *n* = 25; *Lis1^–/+^ n* = 23; *MeCP2^–/y^ n* = 13; double *n* = 9. X^2^ likelihood-ratio (12) = 52.263; *p* = 0.000; *φ* = 0.785; *p* = 0.000]. General locomotion, assessed in the home cage, indicated that only the double mutant mice were significantly less motile than the wild type in the dark active phase [Figure 3B, WT *n* = 25; *Lis1^–/+^ n* = 23; *MeCP2^–/y^ n* = 13; double *n* = 9. X^2^(3) = 7.890; *p* = 0.048; Mean ranks: *MeCP2^–/y^* = 39.73; WT = 38.28; *Lis1^–/+^* = 36.98; double = 17.89. Dunn’s corrected pair-wise comparisons of each group to WT: WT > *double*; *p* = 0.030. WT∼ *Lis1^–/+^*, *p* = 1.000; WT∼ *MeCP2^–/y^*, *p* = 1.000]. To better delineate the motor phenotype of reduced activity, motor coordination and balance were assessed by the mice performance on the beam-walking task (Figure 3C), the CatWalk (Noldus) assessed gait and stride indices (Figures 3D–G). Overall, the trend in the Beam and CatWalk assays is that the double mutants exhibit an exacerbated deviation from the wild-type phenotype as compared with either the *MeCP2^–/y^* and the *Lis1^–/+^* lines.

In the Rotarod assay (Figures 3H,I), that assess cerebellar dependent motor learning, the area under the curve data indicated that the double mutant mice scores did not differ from wild-type mice, yet they performed worse than *Lis1^–/+^* as did the *MeCP2* mice [χ^2^_(3)_ = 14.938; *p* = 0.002; mean ranks: *Lis1^–/+^*(*n* = 6) = 26.33, wt (*n* = 6) = 17.33, double (*n* = 9) = 13.78, *MeCP2* (*n* = 9) = 8.78; Dunn’s multiple pair-wise comparisons: *Lis1^–/+^* > *MeCP2* (*p* = 0.000) and double (*p* = 0.041), all other comparisons *p* > 0.05].

## Discussion

Here, we novel nuclear localization and function for LIS1. These findings change the current concept where LIS1 has been viewed mainly as a molecule regulating the molecular motor, cytoplasmic dynein. Using an unbiased biochemical approach, Histone H1 was defined as a nuclear LIS1 interacting protein, then we further expanded these studies to include an important neuronal histone H1-like molecule MeCP2. Our studies indicate a novel physical and genetic interaction between LIS1 and MeCP2 which affects the interaction between MeCP2 and chromatin, changes gene expression, and modulates adult animal behavior.

The interaction domain between LIS1 and MeCP2, mapped to amino acids 132–288 in the MeCP2 protein. It has been previously noted that Rett syndrome causative mutations are not uniformly distributed all over the protein but rather cluster within the MBD (Nan et al., 1993) and the NCoR/SMRT interaction domains (Lyst et al., 2013), with mutation clusters located in amino acids 97–161 and 302–306. The NCoR/SMRT interaction domain involves direct binding of MeCP2 to transducin beta-like 1 (TBL1) and TBL1 related (TBLR1), two paralogs that are core components of NCoR/SMRT (Kruusvee et al., 2017). TBL1 and TBLR1 are WD repeat proteins similar to LIS1. Recently, MeCP2 KO mice in which a minimal truncated MeCP2 protein was conditionally expressed, were found to exhibit reduced neurological phenotypes and extended survival (Tillotson et al., 2017). The minimal protein included the first 29 N-terminal amino acids from isoform e1, residues 72–173 (MBD) and residues 272–312 (NCoR/SMRT) (Tillotson et al., 2017), these domains overlap in part with the newly designated LIS1 interaction domain. We have also shown that the interaction between LIS1 and MeCP2 is markedly reduced due to mutations in amino acids 133 or 158, which are within the LIS1 binding domain and are among the most frequent detected mutations (4.56 or 8.81%, respectively, Krishnaraj et al., 2017). Therefore, we propose that the interaction between MeCP2 and LIS1 may also consist part of the observed restoration of neuronal functions in the MeCP2 KO mice, following expression of this minimal truncated MeCP2 protein.

**FIGURE 4:**
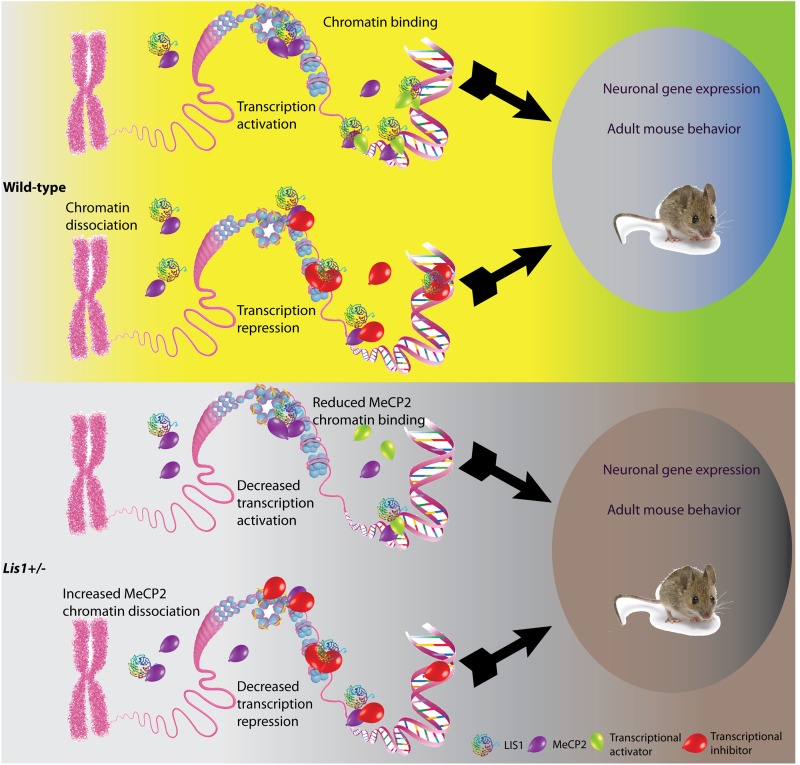
Schematic model illustrating the possible outcomes of LIS1-MeCP2 interaction in the context of wild-type or *Lis1*±. LIS1, shown as a rainbow colored beta-propeller protein, MeCP2, shown as purple balloons, transcriptional activators and inhibitors (green and red balloons, respectively on the bottom), interact in the nucleoplasm, or in close vicinity to the DNA. Transcriptional activation or repression will result in changes in neuronal gene expression as well as adult mouse behavior. **(Top)** In the wild-type context, LIS1 can either bind to MeCP2 in close vicinity to the DNA, and either recruit transcriptional activators, or assist in MeCP2 binding to chromatin. In addition, LIS1 may bind to MeCP2 in the nucleoplasm. These interactions will result in transcriptional activation. In other loci, LIS1 can bind to MeCP2 in close vicinity to the DNA in association with transcriptional repressors, alternatively LIS1 may aid in recruitment of transcriptional repressors to MeCP2. An additional option may be that LIS1 interacts directly with transcriptional repressors and enhances their activity. These interactions will result in transcription repression. **(Bottom)** In the context of *Lis1*±, we can observe reduced LIS1 levels. The effect of LIS1 reduction will be dependent upon the affinity of the different protein interactions. In case of transcription activation, we expect that less LIS1 will be found in association with MeCP2 and transcriptional activators thus resulting in decreased transcription activation. In case of transcription repression, less LIS1 may results in increased levels of MeCP2 in the nucleoplasm and less in association with chromatin.

Our FRAP experiments indicated that the interaction between MeCP2 and chromatin is modulated by the levels of LIS1. LIS1 and MeCP2 are ubiquitously expressed genes, yet their levels of expression vary between different tissues and cell types (Reiner et al., 1995; Song et al., 2014), therefore it is possible that the functional results may differ in a cell type specific manner. In our gene expression studies conducted in primary cortical neurons, we observed massive gene expression changes following either half dosage of LIS1 (*Lis1*±*)* or knockout of MeCP2, with a very high proportion of shared genes. The finding that more than 90% of the transcriptome alterations in MeCP2 knockout neurons were found also in LIS1 heterozygous neurons while on the opposite way it is ∼50% suggests a dependence of MeCP2 function on LIS1. MeCP2 can regulate gene expression at (at least) two different levels: MeCP2 may affect global chromatin organization and in addition can act as a specific transcriptional regulator (Della Ragione et al., 2016). As a transcriptional regulator it functions through recruitment of co-repressors or co-activators (Jones et al., 1998; Nan et al., 1998; Ebert et al., 2013; Lyst et al., 2013). Both LIS1 (rainbow colored beta-propeller protein in Figure 4) and MeCP2 (shown in purple in Figure 4) in the nucleus can be found either in close proximity to the DNA or in the nucleoplasm. MeCP2 can directly bind methylated DNA (Lewis et al., 1992), and it can repress transcription by associating for example with histone deacetylases (shown in red in Figure 4) (Jones et al., 1998; Nan et al., 1998). In addition, LIS1-MeCP2 can activate gene transcription via associated transcriptional activators (shown in green in Figure 4), such as cAMP response element-binding protein 1 (CREB1) (Chahrour et al., 2008). In our model, we depicted how reduced levels of LIS1 may affect MeCP2 activity. The resulting activity will be locus dependent, varies when MeCP2 acts as a transcriptional activator or a transcriptional repressor, and in additional will depend on the affinities of the different protein-protein interactions. Taken together with the massive overlap between MeCP2 affected genes and LIS1 affected genes it seems that LIS1 is involved in both mechanisms of action of MeCP2. It also seems that LIS1 can affect transcription in MeCP2 independent-way probably through its interactions with additional transcriptional factors, which are yet to be identified.

A prominent and previously unappreciated role of LIS1 in transcription regulation was detected in the gene expression studies. Our RNA-seq data demonstrates a very high level of identical DE target genes in neurons derived from the three mutated genotypes in comparison to wild-type. In primary neurons several key pathways associated with synaptic functions were highlighted as being most significantly affected. Differential gene expression was also related to disease phenotypes such as progressive neurological disorders, seizures and epilepsy. These disease phenotypes are relevant to both lissencephaly and Rett syndrome. The changes in gene expression are likely to affect not only neuronal gene expression, but also adult mouse behavior (Figure 4). Indeed, we could observe that the relationships between LIS1 and MeCP2 were also manifested at the level of adult animal behavior although the LIS1 protein was not significantly reduced. Therefore, we conclude that even a slight reduction in LIS1 levels can affect behaviors. All the mutant animal exhibited abnormal nesting properties. The double mutant mice exhibited the worse scores in home cage locomotion, beam walk and run duration. The single MeCP2 mutant mice and the double exhibited reduced speed and decreased stride length in the CatWalk. Interestingly, in the rotarod, a cerebellar dependent assay, the double mutant mice exhibited slightly improved performance than MeCP2 mutant mice, while the heterozygotes *Lis1* exhibited the best performance. Thus, suggesting that LIS1 reduction may alleviate some specific features in relation to mouse behavior. We cannot attribute this specific change to a particular cell type or group of genes, however, this change is likely to result from the physical and genetic interactions between these two genes and their product. Future studies and technologies which may reveal single cell proteomics and protein interactions may assist in explaining this intricate network.

## Materials and Methods

### Animals

This study was carried out in accordance with the principles of the Basel Declaration and recommendations of the Animal Welfare Law (Experiment with animals), The Regulation of the Council for experiments with Animals, The Weizmann Institute Regulations (SOP), The Guide for the Care and Use of Lab Animals, National Research Council, 8th edition, The Guidelines for the Care and Use of Mammals in Neuroscience and Behavioral Research, the Institutional Animal Care and Use Committee of the Weizmann Institute of Science. The protocol was approved by the Institutional Animal Care and Use Committee of the Weizmann Institute of Science.

To study the interaction between LIS1 and MeCP2, *Lis1*±, *MeCP2y/−*, and double-conditionally deleted mice were generated. Mice carrying one floxed allele for *Lis1* gene (129S-*Pafah1b1tm2Awb*/J, Jackson Laboratories) were crossed with mice carrying tamoxifen induced Cre under ubiquitin promoter [B6.Cg-Tg(UBC:cre/ERT2)1Ejb/1J, Jackson Laboratories] resulting in inducible *Lis1* Conditional adult heterozygotes mice. *MeCP2* flox/flox (B6;129P2-*Mecp2tm1Bird*/J, Jackson Laboratories) were crossed with B6.Cg-Tg(UBC:cre/ERT2)1Ejb/1J) mice to generate *MeCP2*-conditionally knockout mice. In the same manner, conditional double mutant mice (*Lis1*flox/wt, *MeCP2* flox/Y) were generated. The double mutant male mice, were generated by crossing the homozygote female of the *MeCP2* flox/flox with homozygote male *Lis1*flox/flox, where both parents expressed the tamoxifen induced Cre (UBC:Cre/ERT2). These mice were subjected to a daily tamoxifen administration to induce the deletion (125 mg of tamoxifen in corn oil per kg of body weight, injected i.p. for 5 consecutive days). The parental lines and the intermediate strains are summarized in the following table.

**Table:** 

**Mice strain**	**Description**
B6.Cg-Tg(UBC::cre/ERT2)lEjb/1J	Inducible, Cre expressed in all tissues following tamoxifen administration
*129S-Pafah1b1^*tm2Awb*^/J*	*Lis1^*flox/flox*^, Lis1* floxed allele
*B6;129P2-Mecp2^*tm1Bird*^/J*	*MeCP2^,]m!^’^]0X^, Mecp2* floxed allele
*129S-Pafah1b1’^*m2Awb*^/J;* B6.Cg-Tg(UBC::cre/ERT2)1Ejb/1J	*Lisl* (exons 3-6) deletion in one allele in all tissues following tamoxifen.
*B6;129P2-Mecp2^*tmBird!*^/J;* B6.Cg-Tg(UBC::cre/ERT2)1Ejb/1J	*Mecp2* (exons 3-4) deletion in all tissues following tamoxifen.
*129S-Pafah1b1^*trn2Awb*^/J;* B6;129P2-*Mecp2^*tm1Bird*^/J;* B6.Cg-Tg(UBC::cre/ERT2)1Ejb/1J	Double mutants, *Lis1* reduction and *Mecp2* deletion following tamoxifen

### Antibodies

Mouse anti-LIS1 antibodies (338) (Sapir et al., 1997) were used for WB (1:1000), for immunoprecipitation (20 ml per sample), and for immunostaining (1:200). Mouse anti-MeCP2 antibodies (Sigma, M6818) were used for WB (1:1000) and for immunoprecipitation (8 ml per sample), chicken anti-MeCP2 antibodies (Millipore, ABE171) were used for WB (1:1000).

### MeCP2 Truncated Proteins Cloning

MeCP2 truncated proteins were cloned and expressed in BL-21 bacteria cells. The following MeCP2 truncated sequences were amplified by a common PCR (using vector contained the mouse e1-MeCP2 sequence), digested by *Eco*RI and Not1 and inserted into pGex 4T-3 vector to generate GST-MeCP2 recombinant proteins. The truncated MeCP2 were amplified using the following primers:

(1) N ter (1–77 + 3 bp): 231 bp
  F: AGCGAATTCTatggtagctgggatgttagg
  R: AGCGCGGCCGCggcttctggcactgctgggg
(2) MBD (3bp + 78–162 + 3 bp): 258 bp
  F: AGCGAATTCTgcctcggcttcccccaaaca
  R: AGCGCGGCCGCccctctcccagttaccgtga
(3) 3 hooks (162 + 365): 609 bp
  F: AGCGAATTCTgggagcccctccaggagaga
  R: AGCGCGGCCGCctccttcttaggtggggagg
(4) TRD (3 bp + 207–310 + 3 bp): 315 bp
  F: AGCGAATTCTggtgttcaggtgaaaagggt
  R: AGCGCGGCCGCcgtctcccgggtcttgcgct
(5) C TER (3 bp + 311–484): 522 bp
  F: AGCGAATTCTgagacggtcagcatcgaggt
  R: AGCGCGGCCGCtcagctaactctctcggtca
(6) N TER + MBD + ID (1–206 + 3 bp): 621 bp
  F: AGCGAATTCTatggtagctgggatgttagg
  R: AGCGCGGCCGCaacaccttctgatgctgctg
(7) MeCP2 (132–208 a.a): 253 bp
  F: AGCGAATTCTtttcgctctaaagtagaatt
  R: AGCGCGGCCGCctgaacaccttctgatgctg
(8) MeCP2 (207–288 a.a): 268 bp
  F: AGCGAATTCTgttcaggtgaaaagggtcct
  R: AGCGCGGCCGCcacggctttctttttggcct
(9) MeCP2 (132–288 a.a): 492 bp
  F: AGCGAATTCTtttcgctctaaagtagaatt
  R:AGCGCGGCCGCcacggctttctttttggcct

### GST Tagged Protein Purification

GST-fusion plasmids, representing different MeCP2 fragments and MeCP2 full length, were transformed into BL21 (DE3) RIL bacteria (Stratagene, La Jolla, CA, United States), which were grown in LB at 37°C to an optical density of 0.7–0.8. Induction of protein expression was carried out using 0.2 mM IPTG for 3 h or 0.05 Mm IPTG over night at 17°C (when protein was seen in the pellet). Protein was extracted in NETN buffer (0.5% Nonidet P-40, 0.1 M NaCl, 1 mM EDTA, 20 mM Tris-HCl, 0.1mg PMSF, pH 8.0) with sonication. The soluble fraction was bound to glutathione agarose beads (Sigma, Rehovot, Israel) for 1 h, cleared by centrifugation above 20% sucrose–NETN, and washed extensively in NETN buffer before elution from column in 50 mM Tris-HCl, pH 8.0, 0.15 M NaCl, 10 mM glutathione and 10% glycerol.

### Far Western

Far-Western analysis was conducted as previously described (Sato et al., 2011). Briefly, total extracts taken from induced BL-21 bacterial cells that express the recombinant truncated proteins were separated in SDS-PAGE and transferred to a nitrocellulose membrane. The proteins in the membrane were then denatured with guanidine and renatured with gradient-reducing guanidine, which allows proteins to recover their structure and the membrane was blocked in protein-binding buffer [100 mM NaCl, 20 mM Tris-HCl (pH 7.5), 0.5 mM EDTA, 10% glycerol, 0.1% Tween-20, 2% skim milk powder, 1 mM DTT] at 4°C for overnight. Next, the membrane was probed with 35S-methionone labeled LIS1 in 2 ml of protein-binding buffer in shaking at 4°C for overnight. The probe was generated *in vitro* using the TNT T7 Quick Coupled Transcription/Translation System following the manufacturer’s protocol (Promega, L1171). Following three washes with the protein-binding buffer, the membrane was dried and exposed to a phosphorimager screen.

### 6X His Tagged Protein Purification

Recombinant LIS1 prepared in insect cells was purified as described in the QIAGEN handbook for high level expression and purification of 6 His-tagged proteins. Protein was extracted in lysis buffer (50 mM NaH2PO4, 300 mM NaCl, 10 mM imidazole, pH 8.0) with sonication. The soluble fraction was bound to Ni-NTA slurry (MCLAB, NINTA-200) for 1 h at 4°C, and washed extensively in wash buffer (50 mM NaH2PO4, 300 mM NaCl, 20 mM imidazole, pH 8.0) before elution from column in 50 mM NaH2PO4, 300 mM NaCl, 250 mM imidazole, pH 8.0.

### Microscale Thermophoresis (MST)

This technique allows measuring the affinity of the molecular interactions under close-to native conditions. MST is based on detection of changes in the hydration shell, charge or size of molecules. An infrared-laser is used to generate precise microscopic temperature gradients within thin glass capillaries that are filled with a sample in a buffer or bioliquid of choice. The fluorescence of molecules is used to monitor the motion of molecules along these temperature gradients. Prior to the experiments all the recombinant proteins were dialyzed three times against >200x volume of 0.33 × PBS at 4°C. 6XHis-LIS1 protein was fluorescently labeled using the Monolith NT Protein Labeling Kit RED according to the manufacturer protocol. Labeled 6X His-LIS1 used at a concentration of 2.5 mg/ml was incubated with increasing concentrations of recombinant GST-tagged WT MeCP2 or the recombinant mutated MeCP2 proteins (R-C a.a 133 or T-A a.a 158). The experiments were performed in binding buffer [150 mM NaCl, 20 mM Tris-HCl (pH 7.5), 0.5 mM EDTA, 10% glycerol, 0.1% TWEEN 20, 1 mM DTT] and measured in standard treated capillaries. All binding reactions were first incubated for 10 min at room temperature before they were loaded into the capillaries. The measurements were done on a NanoTemper Monolith NT.115 instrument. All measurements were performed at 50% LED and 80% IR-Laser, Laser-On time was 30 s, Laser-Off time 5 s.

### Production of Adenoviruses

AdEasy XL Adenoviral Vector System (Agilent) was used for production of adenovirus expressing GFP-fused MeCP2 according to the manufacturer protocol. Cortical primary neurons infected by this MeCP2 fluorescent virus were used in the FRAP experiment.

### Cortical Neuronal Culture

P0 mouse pups heads were passed in 70% ethanol for disinfection. Then, cortices were isolated in ice-cold L-15 media supplemented with glucose (0.6%), bubbled with 95% O2 and filter-sterilized. The forebrains were dissected and the meningeal membranes and blood vessels were removed. Telenchephalic cortices were dissected and incubated for 30 min at 37°C in HBSS supplemented with 0.6% glucose, gentamicin (20 μg/ml; Sigma), 0.25% trypsin, and 0.75 mg/ml DNAse I. Next, the tissue was mechanically dissociated in neuronal media [MEM supplemented with 0.1 mg/ml gentamicin (Sigma), 2 mM L-Glutamax, 5% HS, 5% FCS, 2xB27 (1% Gibco 0153)]. Dissociated cells were plated on plates pre-coated with poly-L-lysine (0.04% sigma, P4707) and laminin (0.01%, Sigma, L2020) in neuronal medium (MEM) supplemented with 0.1 mg/ml gentamicin (Sigma), 2 mM L-Glutamax, 5% HS, 5% FCS, 2xB27 (1% Gibco 0153). Following 4 days, 20 μg/ml FUDR (Sigma, F0503) was added for the elimination of glial cells.

### Fluorescence Recovery After Photobleaching

*Lis1*± and WT cortical neurons plated on MaTek plates pre-coated with poly L-lysine (0.04% Sigma, P4707) and laminin (0.01% Sigma, L2020) were infected with adenoviruses expressing MeCP2-GFP 72 h before the FRAP experiment. FRAP experiment was conducted with Zeiss 800 LSM confocal microscope according to the detailed explanation found in https://www.youtube.com/watch?v=EoGj7q7vUbU. (WT cortical neuronal cell, *n* = 21; *Lis1*±, *n* = 24). The recovery time was calculated (WT cortical cell, *n* = 21; *Lis1*± cortical cells, *n* = 24). The experiment was repeated three times, and statistical analysis was determined using the Student’s *t*-test.

### Behavioral Assays

At the age of 12 weeks mice were injected with tamoxifen for 5 days and after 3 weeks were placed in a reversed 12 h light and dark cycle room. The behavioral tests were conducted on males 1 month following the tamoxifen treatment (16 weeks of age).

All behavioral studies were performed during the dark active phase period. Mice were habituated to the test room for 1 h before each test. In between assays mice recovered for at least 2 days.

#### Nesting

The test was performed as previously described (Deacon, 2006). Briefly, 1 h before the dark phase, the mice were transferred to individual testing cages with one cube of Nestlet. Following 13 h, in the light phase, the degree of nest building was assessed on a scale of 0 – 4, where 0 was no nest and 4 was when the mouse generated a very nice round and high nest.

#### Home Cage Locomotion Adapted From Panayotis et al. (2018) (InfraMot; TSE-Systems, Bad Homburg, Germany)

This home-cage based movement-quantifying system registers activity by sensing the body-heat image (infra-red radiation) and its spatial displacement over time. The mice were single caged for a period of 72 h. The first 24 h were considered as an habituation period and the collected data includes counts of changes in the body-heat image that represent movement of the mouse over two dark-light cycles (48 h). The mean hourly activity during the dark, active, phase was used as an index of voluntary motility.

#### Balance Beam (Adapted From Appel et al., 2016)

The Balance beam assay assesses balance and coordination. In this test, mice were trained to walk on a beam (500 mm long) in order to return to their home cage. In the first day mice were trained, over five trials, using a wide beam (30 mm); the next day they were required to cross a narrow (5 mm) beam. Each test session consisted of five consecutive trials. The latency to cross the beam is recorded and the mean of these five test trials was used as an index.

#### CatWalk (Noldus, Wageningen, Netherlands) Adapted From Perry et al. (2012)

This system was used for quantitative assessment of footfalls and gait. The apparatus consists of an enclosed walkway that a mouse walks on; the “Illuminated Footprints” technology allows a high-speed video camera (positioned underneath the walkway) to capture the footprints. These images are processed based on the dimensions, position and dynamics of each footfall to produce quantitative analyses of footfalls and gait: Each mouse went through a test session that was comprised of five “runs” (the mouse walks the full length of a 50 cm runway) that comply with minimal speed variation requirements (less than 60%). The following indices were analyzed: run duration, mean speed, stride length (front and hind legs separately) and base of support, i.e., the distance between girdle paws (front and hind legs separately).

#### Rotarod (Rotor Rod System, San Diego Instruments, San Deigo, CA, United States) Adapted From Ezra-Nevo et al. (2018)

The assay is useful in assessing sensorimotor functions of balance and coordination. In this procedure mice were trained for 5 days to walk on a rotating drum. The test session is comprised of five trials for each mouse (inter-trials-interval: at least 1 min); all trials are acceleration trials (0–40 RPM in 4 min) and the latency to fall was recorded. The first two trials were regarded as habituation and the mean of the last three trials was used as the score.

### RNA Sequencing

Total RNA was extracted using the RNeasy Mini kit (cat. No. 74104, Qiagen) following the manufacturer’s protocol. RNA concentration (Nanodrop, Thermo Scientific) was measured followed by validation of integrity by the Agilent Tapestation. Libraries were prepared from 50 ng of total RNA using Bulk MARS-seq to produce expression libraries with a minimum of three replicates per group as described previously (Jaitin et al., 2014). The quality of the libraries was assessed by tapestation and qPCR, and high-quality libraries were sequenced by the Illumina NextSeq 500 sequencer to obtain single reads of 75 bp. RNA concentration and integrity were measured using a Thermo Scientific Nanodrop 2000 spectrophotometer and an Agilent Tapestation 2200, respectively. RNA samples with RIN > 7 were taken for library prep.

### RNA Differential Expression Analysis

Reads were trimmed with Cutadapt (Martin, 2011) and mapped to the mouse genome GRCm38/mm10 using STAR v2.4.2a (Dobin et al., 2013) with the parameters:alignEndsType EndToEnd; alignSoftClipAtReferenceEnds No and outFilterMismatchNoverLmax 0.05. After collapsing UMIs, we used the 3′ end (1000 bp) of GENCODE transcripts (Frankish et al., 2018) for counting the number of reads per gene using HTSeq-count in union mode (Anders et al., 2015). Further analyses were done in R (version 3.5.1). Normalization and test of differential expression analysis was performed using DESeq2 (Love et al., 2014) with lfcShrink = normal for estimating log2 fold changes. Raw *p*-values were adjusted for multiple testing using Benjamini-Hochberg. We set the threshold for significant differentially expressed genes to: log2 fold change > 1 or < −1, adjusted *p*-value < 0.05 and an averaged normalized count of ≥30. Gene ontology analysis was conducted using the clusterProfiler package (Yu et al., 2012) with adjusted *P*-values (Benjamini and Hochberg method) cutoff 0.05. Venn diagrams and heatmaps were plotted with eulerr package (Larsson, 2018) and pheatmap package (Kolde, 2018), respectively.

### Co-chIP of LIS1 and MeCP2 (From Mouse P1 Brains)

P1 fresh brains were perfused with 1% PFA, removed and incubated with 122 Mm Glycine three times for 5 min, each and washed with PBS. Brain regions were suspended in Lysis buffer (20 mM HEPES pH 7.8, 10 mM KCl, 2 mM MgCl2, 10 mM NaF, 0.05% NP40 and protease inhibitor cocktail) and lysed by dounce homogenizator (seven strokes each). After centrifugation (10 min at 500 *g* at 4°C), the pellet was resuspended in RIPA buffer supplemented with protease inhibitor cocktail × 100, incubated on ice for 10 min and sonicated (13 times, 1 s on, 1 s off, 2 min on ice in between the cycles). Next, the samples were cleared by centrifugation for 10 min, 20000 *g* at 4°C. For immunoprecipitation, A/G protein beads were incubated with Rabbit anti-MeCP2 antibodies (8 μl for each sample) or with Goat anti-LIS1 antibodies (30 μl for each sample) in blocking buffer (1xPBS, 0.5% Tween 20, 0.5% BSA) in rotation for 1h at RT. Next, the lysate was added to the beads-antibodies solution and incubated in rotation during an overnight at 4°C. Immunoprecipitated proteins were pelleted by centrifugation, washed 6 times with RIPA buffer, eluted by boiling in SDS-PAGE sample buffer for 35 min, and analyzed by Western blot analysis.

#### Co-IP (Immunoprecipitation) of LIS1 and MeCP2 (From Mouse P1 Nuclear Extracts)

Immunoprecipitation was performed from lysates prepared from P1 WT pups. Nuclear brain extracts were prepared in two steps of lysis to separate the cytoplasmic and the nuclear fractions. Brains were suspended in buffer A (10 mM HEPES pH 7.8, 10 mM KCl, 2 mM MgCl2, 10 mM NaF, 0.05% NP40 and protease inhibitor cocktail) and lysed by a dounce homogenizator (nine strokes each). After centrifugation (for 5 min at 500 *g* at 4°C) the pellet (the nuclear fraction) was resuspended in 1.5 volumes of high salt buffer (20 mM HEPES pH 7.8, 0.6 M KCl, 2 mM MgCl2, 25% glycerol, 10 mM NaF, protease inhibitor cocktail), incubated on ice for 30 min and centrifuged for 30 min at 24000 *g* at 4°C. For immunoprecipitation, A/G protein beads were incubated with anti-MeCP2 antibodies (8 μl for each sample, M6818) or with anti-LIS1 antibodies (20 μl for each sample, 338) in blocking buffer (1xPBS, 0.5% Tween 20, 0.5% BSA) in rotation for 1h at RT. Next, the nuclear lysate was added to the beads-antibodies solution and incubated in rotation overnight at 4°C with IP buffer (50 mM Tris-HCl pH 7.5, 150 mM NaCl, 1% Triton X-100 and protease inhibitor cocktail). Immunoprecipitated proteins were pelleted by centrifugation, washed three times with IP buffer, eluted by addition of SDS-PAGE sample buffer, boiled for 10 min, and analyzed by Western blot analysis.

#### Immunostaining

HeLa JW cells plated on cover slips were fixed in 3% paraformaldehyde in PHEM buffer (60 mM PIPES, pH 6.9, 25 mM HEPES, 10 mM EGTA, 2 mM magnesium acetate) for 20 min. Following three washings of 5 min each in PBS the cells were permeabilized for 25 min in 0.1% Triton X-100 in PBS. The cells were treated with 50 mM NH_4_Cl in PBS for 10 min, washed three times for 5 min each in PBS, blocked in PBS-BSA (PBS containing 0.1% BSA), and labeled with anti-LIS1 mouse monoclonal antibodies (210) (Sapir et al., 1997) and anti-Lamin B gout antibodies (SC-6216, Santa-Cruz) for 1 h at 37°C. Following three washings of 10 min each in PBS-BSA, the cells were incubated with secondary antibodies; Cy3-labeled donkey anti mouse or FITC-labeled donkey anti goat (Jackson ImmunoResearch Laboratories, West Grove, PA, United States) for 45 min at 37°C. The DNA was stained with DAPI (Sigma) for 5 min. The coverslips were mounted using Vectashield^®^ (Vector Laboratories, Burlingame, CA, United States). Pictures of single planes were collected using confocal laser-scanning microscope (Bio-Rad Radiance 2100, mounted on Nikon TE300).

#### Statistical Analysis

Statistical analysis was conducted using Prism 5 and 7 for Mac OS X using *Student’s t-test* or ANOVA as appropriate for the assay.

## Data Availability

RNA-seq data has been deposited in NCBI with the accession number of GSE124521.

## Ethics Statement

The protocol was approved by the Institutional Animal Care and Use Committee of the Weizmann Institute of Science.

## Author Contributions

LK planned and conducted the experiments, analyzed the data, and participated in writing the manuscript. GG planned and conducted the experiments, conducted all the linker histone related experiments, set the conditions for the FRAP analysis, analyzed the data, and participated in writing the manuscript. AK planned and conducted the RNA seq experiments and related data analysis, prepared figures, and participated in writing the manuscript. MT planned and analyzed behavioral experiments and participated in writing of the manuscript. TO, XW, YY, Y-SC, Y-GY, and IV planned the experiments were involved in RNA seq experiments, data analysis and manuscript writing. OR planned the experiments, analyzed the data, and wrote the manuscript.

## Conflict of Interest Statement

The authors declare that the research was conducted in the absence of any commercial or financial relationships that could be construed as a potential conflict of interest.
