# Supplementary material for: Interplay of LIS1 and MeCP2: Interactions and Implications With the Neurodevelopmental Disorders Lissencephaly and Rett Syndrome
Source: Front Cell Neurosci. 2019 Aug 14;13:370. doi: 10.3389/fncel.2019.00370 (PMC6703185; doi:10.3389/fncel.2019.00370)
Supplement: Supplementary file 1 [file Data_Sheet_1.PDF]

Supplementary Figures

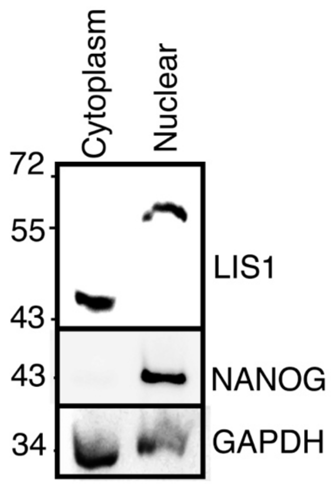

Supplementary Fig. 1: **Cytoplasmic and nuclear fractionation of LIS1.** Mouse embryonic stem cells V6.5 were fractioned for the cytoplasm and the nucleus. The protein lysates were separated by PAGE and blotted with anti-LIS1 antibodies, showing immunoreactivity in the cytoplasm and the nucleus, Nanog, which is exclusively nuclear, and GAPDH, while most is in the cytoplasm, is in part in the nucleus.

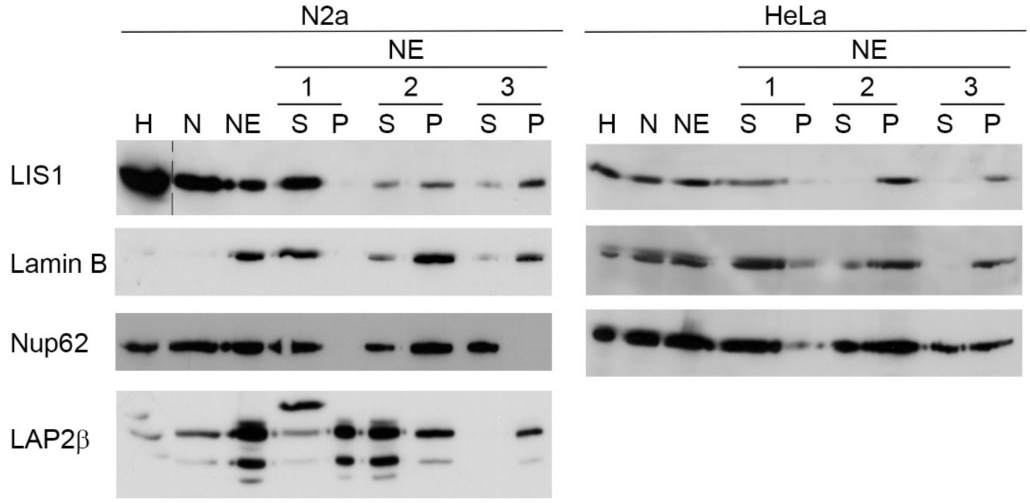

Supplementary Fig. 2: **Nuclear and nuclear envelope localization of LIS1.** Whole cell homogenate (30 µg) (H), isolated nuclei (N), isolated nuclear envelopes (NE), supernatants (S) and pellets (P) of sub-fractions of nuclear envelope (1-3) from HeLa and N2a cells were separated by SDS-PAGE. Nuclear envelope sub-fractions were generated by treating 30 µg with urea and sodium carbonate to solubilize the nuclear lamina and the nuclear pore complexes (1), Triton-X100 and SDS to solubilize the nuclear membranes (2) and Empigen BB on the pellet generated by the second treatment to solubilize the nuclear pore complexes (3). Western blot

analysis was carried out with antibodies against LIS1, Lamin B (nuclear lamina marker), LAP2β (nuclear membrane marker) and Nups (Ab414 against nuclear pore complex proteins, Nup62 is presented).

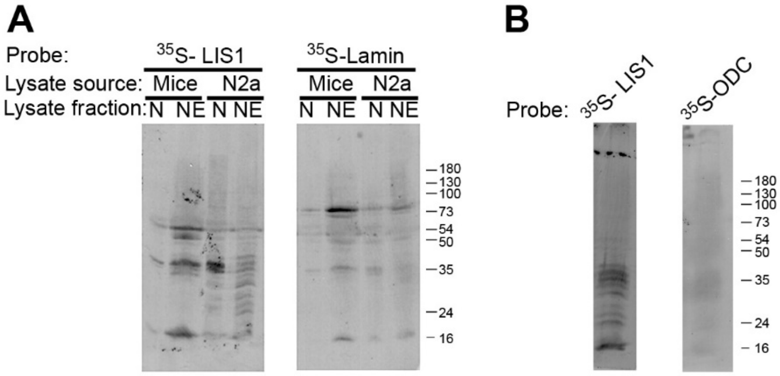

Supplementary Fig. 3: **LIS1 interacts with nuclear and nuclear envelope proteins.**

A) Nuclei and nuclear envelopes isolated from mice brains (7.5 μg of protein extract) or N2a cells (50 μg of protein extract) were separated by SDS-PAGE, blotted to nitrocellulose membrane and incubated *in vitro* translated ^{35}S-labeled LIS1 or Lamin probes with equal specific activity. The blots were exposed for the same time to an X-ray film. B) Nuclear envelope protein extracts (100 μg) were separated by SDS-PAGE, blotted to nitrocellulose membranes and incubated with *in vitro* translated ^{35}S-labeled LIS1 or ODC probes of equal specific activity. The blots were exposed for the same time to an X-ray film. The protein ladder marks are in KDa.

A

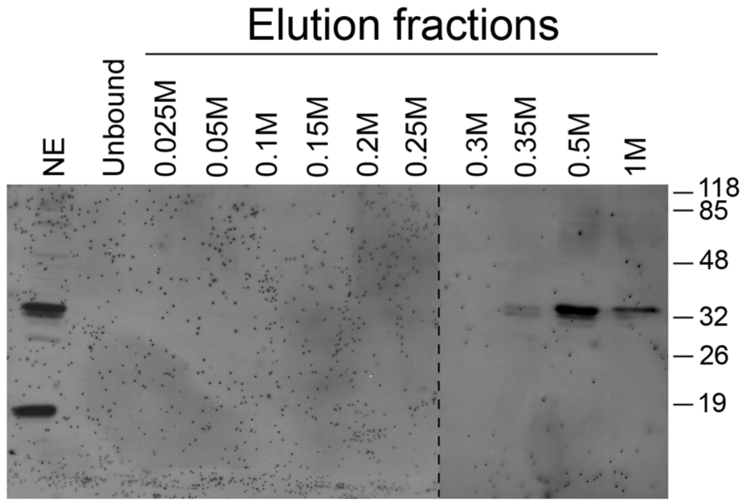

B

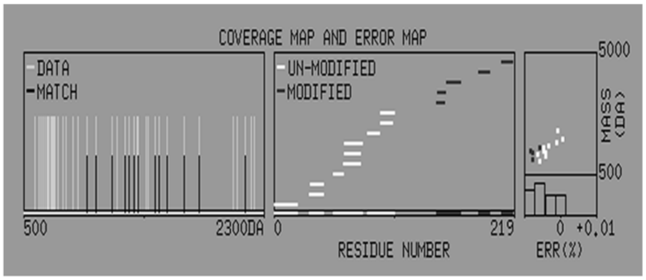

Measured Mass(M)	Avg/ Mono	Computed Mass	Error (%)	Residues Start	To	Missed Cut	Peptide sequence
972.532	M	972.596	-0.007	55	64	1	SGVSLAALKK
1045.532	M	1045.625	-0.009	149	156	3	KSTKKTPK (3)+CH3CO@K;
1045.532	M	1045.625	-0.009	150	157	3	STKKTPKK (3)+CH3CO@K;
1165.552	M	1165.609	-0.005	98	109	1	GTGASGSFKLNK
1259.652	M	1259.744	-0.007	86	97	1	SLVSKGTLVQTK
1293.642	M	1293.703	-0.005	98	110	2	GTGASGSFKLNKK
1325.672	M	1325.791	-0.009	187	197	2	SPAKAKTVKPK (4)+CH3CO@K;
1355.692	M	1355.765	-0.005	34	46	1	KTSGPPVSELITK
1355.692	M	1355.825	-0.010	208	218	3	AAKPKKTAAKK (5)+CH3CO@K;
1481.792	M	1481.892	-0.007	158	171	3	AKKPAAAAGAKKAK (4)+CH3CO@K;
1511.782	M	1511.866	-0.006	33	46	2	RKTSGPPVSELITK
1577.722	M	1577.779	-0.004	65	79	1	ALAAAGYDVEKNNSR
1705.852	M	1705.874	-0.001	64	79	2	KALAAAGYDVEKNNSR
1818.972	M	1818.958	0.001	65	81	2	ALAAAGYDVEKNNSRIK
2162.122	M	2162.139	-0.001	1	22	2	MSETAPAAPAAPAPAEKTPVKK

Supplementary Fig. 4: **LIS1 interacts with histone H1E**. B16-F1 purified nuclear envelopes (0.6 mg) (NE) were treated with 5 M NaCl, dialyzed and loaded onto a DEAE column. The proteins were eluted by increasing concentrations of NaCl and were precipitated with ethanol. Twenty-five percent of each of the different eluted fractions and of the unbound fraction and 5% of the dialyzed extract were separated by SDS-PAGE and blotted to a nitrocellulose membrane. Following blocking, *in vitro* translated ^{35}S-labeled LIS1 was added as a probe for an overnight incubation. Next, the membrane was washed and exposed to an X-ray film. The protein ladder marks are in KDa. B. Seventy five percent of the fraction eluted with 0.5 M NaCl was separated by SDS-PAGE, and stained with Coomassie Brilliant Blue. The 35KDa protein was eluted from

the gel and analyzed by MALDI-TOF. The detected masses and their identification as analyzed by ProFound program (Rockefeller University) are presented.

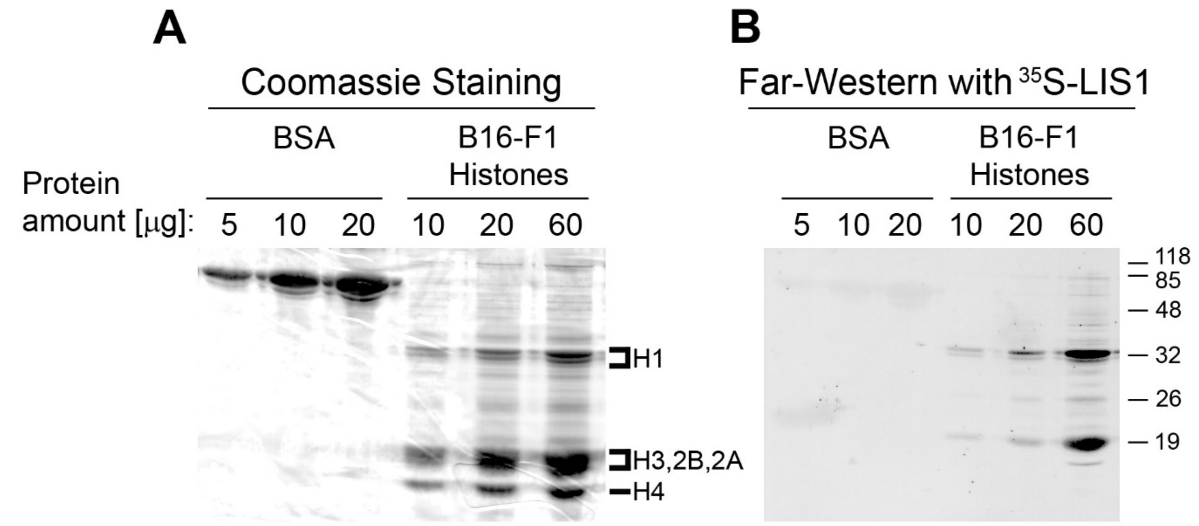

Supplementary Fig. 5: **LIS1 interacts with acid purified histone H1.** Indicated amounts of acid-extracted histones from B16-F1 cells or bovine serum albumin (BSA) were separated in two identical 12% SDS-PAGE. A) One gel was stained with Coomassie Brilliant blue for detection of the general protein level. The different histones are indicated. B) The second gel that was used for Far-Western analysis was blotted onto a nitrocellulose membrane. Following blocking, *in vitro* translated ^{35}S-labeled LIS1 was added as probe for an over-night incubation. Next, the membrane was washed and exposed to an X-ray film. The ~35 KDa band is histone H1.

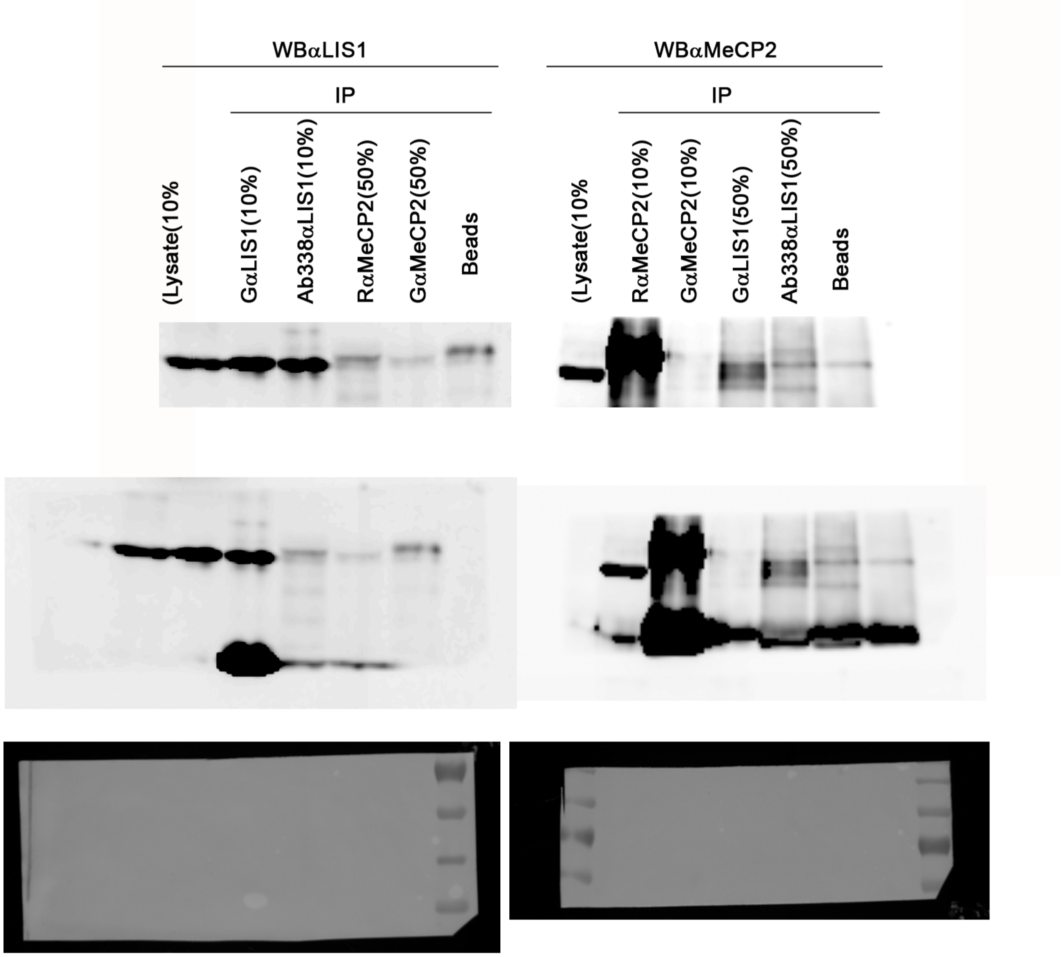

Supplementary Fig. 6: Original blots (uncut) related to figure 1B.

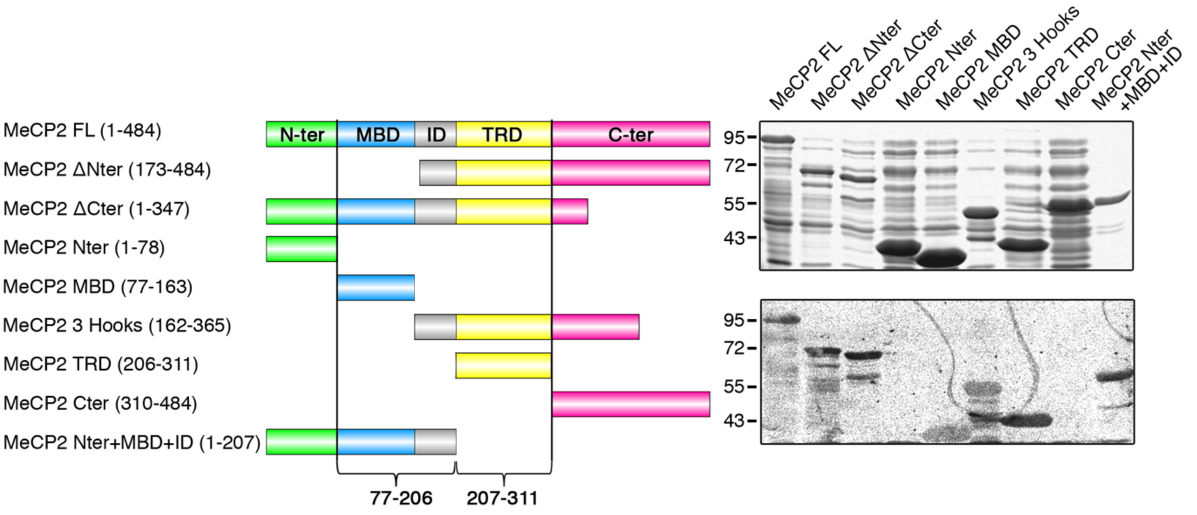

Supplementary Fig. 7: LIS1 interacts directly with MeCP2 in a defined tandem repeat domain *in vitro*. Recombinant full-length (FL) or indicated fragments of MeCP2 were expressed in bacteria. Bacterial lysates were separated by SDS-PAGE, the gels were either Coomassie-blue

stained (top box on right). Far-western analysis (bottom box on right) indicates that LIS1 can bind to FL MeCP2 and to fragments of the protein located between amino acids (a.a.) 77-206 and 207-311.

```
Alignment of Sequence_1:  [MeCP2 77-207.xprt] with  Sequence_2: [MeCP2 207-311.xprt]

Similarity : 55/74 (74.32 %)

Seq_1  1     PEASASPKQRRSIIRDRGPMYDDPTLPEGWTRKLKQRKSGRSAGKYDVYLINPQGKAFRS  60
                                                                       ::
Seq_2  1     ---------------------------------------------------------VQV  3


Seq_1  61    K-VELIAYFEKVGDTSLDPNDFDFTVT--G-RGSPSRREQK-PPKKPKSPKA-P-GTGRG  113
             | | |: :: |:::::::::::: :::  |   | ::: :| | :| |  :| |  ::::
Seq_2  4     KRV-LEKSPGKLLVKMPFQTSPGGKAEGGGATTSTQVMVIKRPGRKRKA-EADPQAIPKK  61


Seq_1  114   RGRPKGSGTTRPKAATSEGV------------------------  133
             ||| :||:  :  |: : ::
Seq_2  62    RGRKPGSVVAAAAAEAKKKAVKESSIRSVQETVLPIKKRKTRET  105
```

Supplementary Fig. 8: **Some internal similarities in MeCP2**. Partial sequence of MeCP2, amino acids 77-207 and 207-311 were aligned. Identical and similar amino acids are indicated.

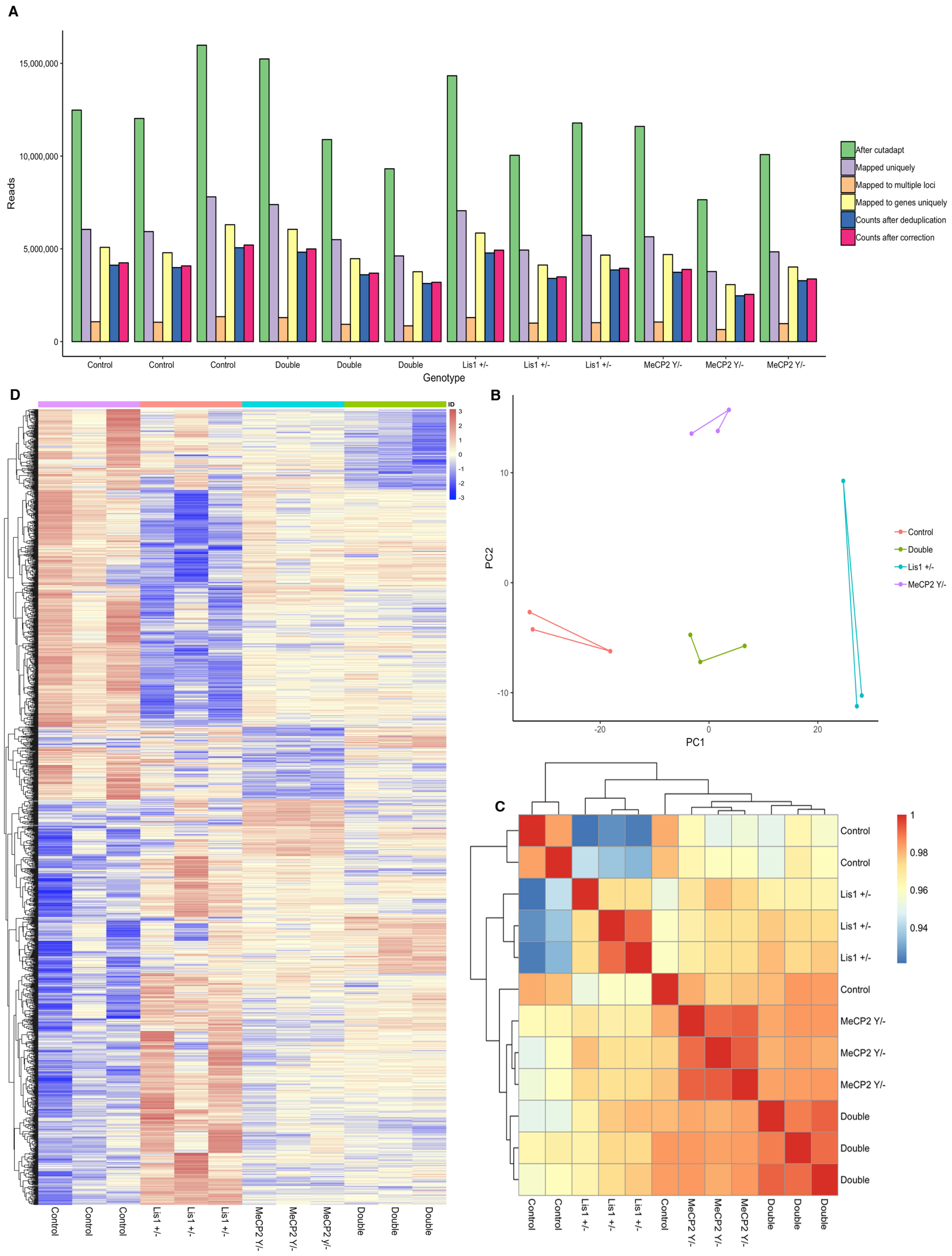
A
B
C
D
Reads
15,000,000
10,000,000
5,000,000
0
Genotype
Control
Double
Lis1 +/-
MeCP2 Y/-
MeCP2 y/-
After cutadapt
Mapped uniquely
Mapped to multiple loci
Mapped to genes uniquely
Counts after deduplication
Counts after correction
PC1
PC2
ID
0.98
0.96
0.94

Supplementary Fig. 9: **RNA-Seq additional information.** A) Histogram with the number of reads considered for all the samples in each sequential step of the RNA sequencing analysis for estimating differentially expressed genes. B) Principal component (PC) analysis of most variable genes, and showing the top two principal components (PC1- 66% variance, PC2-16% variance). C) Hierarchical clustering of Pearson correlation coefficients between 12 samples from four genotypes with three repeats each. D) Hierarchical clustering and heatmap representation of differentially expressed genes exclusive to each comparison i.e. Control vs. Lis1+/-, Control Vs. MeCP2 Y/-, Control vs. Double mutants. The color-coded scale represents the z-score of DESeq2 normalized expression value. Presented genes exhibit log2 fold change value > 1 or < -1, and adjusted p-value < 0.05.

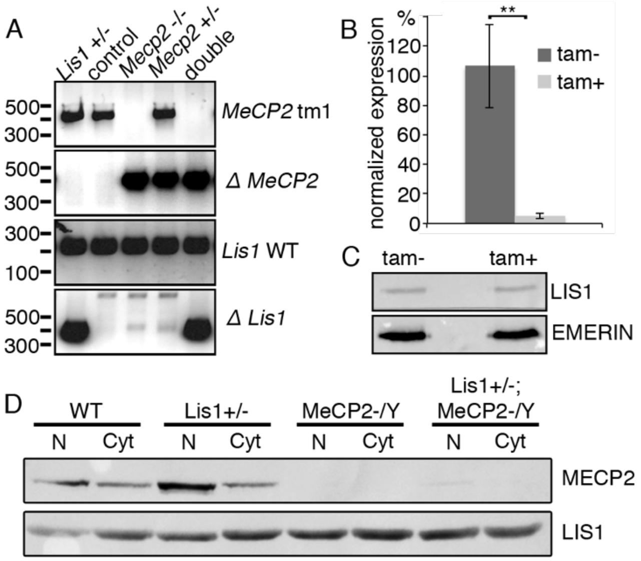

Supplementary Fig. 10: **Deletion of *Lis1* or *MeCP2* alleles.** A) *MeCP2* was deleted in the mice carrying the *MeCP2* tm1 allele or deleted (two top panels). The *Lis1* wild type allele was present in all the mice and the deleted allele was present in the heterozygous. B) *Lis1* RNA relative level in granular neurons derived from P8 homozygotes *Lis1* floxed allele mice treated or non-treated with tamoxifen. A significant reduction in Lis1 mRNA level was observed following 2 weeks of tamoxifen treatment. Data is shown as mean ± SEM. **p < 0.01. C) LIS1 protein level in granular neurons derived from P8 homozygotes *Lis1* floxed allele mice treated or non-treated with tamoxifen for one month in culture. D) LIS1 and MeCP2 protein levels using nuclear (N) and cytoplasmic (Cyt) cerebellar extracts from our mutant behaving mice.

Supplementary Materials and Methods

Sub-cellular fractionation Nuclear envelopes were purified according to Emig et al. [1] cells were homogenized in STM 0.25 buffer (50 mM Tris pH 7.4, 0.25 M sucrose, 5 mM $MgSO_4$, 2 mM DTT, 10 mM β–Glycerophosphate, 10 mM NaF, 1 mM sodium orthovanadate and protease inhibitors) supplemented with 0.025% NP-40. The homogenate was adjusted to 1.4 M sucrose by addition of an appropriate volume of STM 2.1 (as STM 0.25 buffer but with 2.1 M sucrose) and laid between STM 2.1 buffer, STM 0.8 buffer (as STM 0.25 buffer but with 0.8 M sucrose) and STM 0.25 buffer. Followed centrifugation at 100,000 *g* for 65 min, the nuclei pellet was re-

suspended to protein concentration of 0.7 mg/ml in TP buffer (10 mM Tris pH 7.4, 10 mM phosphate buffer pH 7.4, 5 mM $MgSO_4$, 10 mM β–Glycerophosphate, 10 mM NaF, 1 mM sodium orthovanadate and protease inhibitors) containing 0.25 mg/ml heparin and 36 U/ml DNase I. Following shaking for 60 min at 4°C and 15 min at R.T. the nuclear envelopes were pelleted by centrifugation at 10,000 *g* for 30 min. The nuclear envelopes pellet was re-suspended in STM 0.25 buffer and further fractionation was carried out according to Cronshaw et al. [2]. 4M Urea and 0.1M sodium carbonate treatment was used for solubilization of the lamina and the nuclear pore complex fractions. 1% Triton-X100 and 0.025% SDS treatment was used for solubilization of the nuclear membrane fraction. 0.3% Empigen BB treatment of the pellet generated by the second treatment was used for solubilization of the nuclear pore complex fraction.

Antibodies Mouse Ab210 anti LIS1, Goat anti Lamin B (sc-6216, Santa Cruz Biotechnology), mouse Ab414 (a gift from Michael Elbaum, Weizmann Institute of Science) and mouse anti LAP2β (a gift from Amos Simon, Sheba Medical Center).

Histones acid extraction B16-F1 cells were lysed in histone lysis buffer (10 mM Tris-HCl pH 6.5, 50 mM $NaHSO_3$, 10 mM $MgCl_2$, 1% Triton X100, 8.6% Sucrose and 10 mM Na-Butyrate). Following a 5 min incubation on ice the nuclei were precipitated by centrifugation at 10,000 g for 5 min at 4°C. The nuclei pellet was washed three times with histone lysis buffer and once with histone wash buffer (10 mM Tris-HCl pH 7.5 and 13 mM EDTA). Next, the nuclei pellet was re-suspended in water and sulforic acid was added to a final concentration of 0.4 N. Following 1 h incubation on ice the nuclei were centrifuged at 10,000 g for 5 min at 4°C, and the histones containing supernatant was collected. Following acetone precipitation the histones were re-suspended in Tris buffer (20 mM Tris-HCl pH7.4 and 5% glycerol) and kept at –70°C.

Far-Western analysis Far-Western analysis was carried out as previously described [3]. The probes were generated from plasmids expressing human LIS1 [4], Drosophila Lamin (generous gift from Yossi Gruenbaum) [5] and mouse ornithine decarboxylase (ODC) (generous gift from Chaim Kahana) [6] under T7 promoters.

DEAE column 0.2 g of DEAE fibers (DE23, Whatman, Maidstone, UK) were swallowed in 25 ml of 0.5 M HCl for 30 min. Following four washings with water (the pH was increased to 4) the fibers were incubated twice in 25 ml of 0.5 M NaOH for 30 min each. Following three washings in 25 ml of water (the pH reached 7) the fibers were washed once with 10 ml of 1 M Tris-HCl pH 7.5, once with 10 ml of column buffer (20 mM Tris HCl pH 7.5, 10 mM NaCl, 10% Glycerol, 0.5 mM DTT and 1 mM PMSF) and were brought to 50% slurry with the column buffer. Proteins were released from 0.6 mg of nuclear envelopes from B16-F1 by incubation of the nuclear envelopes in 0.2 ml of 5 M NaCl for 30 min at 4°C in rotation. The membranes were precipitated by a 15 min of centrifugation at 10,000 g at 4°C. The supernatant was dialyzed three times against 120 ml of column buffer at 4°C for 1.5 h each. The received proteins were incubated with the DEAE fibers for 3 hr at 4°C in rotation in a final volume of 2.5 ml of column buffer supplemented with 10 μg/ml of leupeptin and 1 μg/ml of pepstatin A. Next, the fibers were precipitated by centrifugation at 1,400 rpm for 1 min in table centrifuge and the supernatant was kept as the unbound fraction. The fibers were re-suspended in 1 ml of column buffer, loaded on a column, washed with 4 ml of column buffer and eluted with step gradient of increasing NaCl concentration (from 0.025 M to 0.5 M) in column buffer supplemented with 10 μg/ml of leupeptin and 1 μg/ml of pepstatin A. The 0.7 ml fractions were precipitated by incubation with 1.3 ml of ethanol at –20°C for an over-night. Following 30 min centrifugation at 10,000 g at 4°C

the protein pellets were dried in Speed-Vac, boiled in 2x sample buffer and 25% of each sample were used for a Far-Western analysis. 75% of the fraction eluted with 0.5 M NaCl were separated in SDS-PAGE, stained with Coomassie and the 35KDa protein was cut out of the gel and was sent for a mass-spectrometry analysis at the Weizmann Institute Proteomics facility.

1 Emig, S., Schmalz, D., Shakibaei, M. & Buchner, K. The nuclear pore complex protein p62 is one of several sialic acid-containing proteins of the nuclear envelope. *J Biol Chem* **270**, 13787-13793 (1995).

2 Cronshaw, J. M., Krutchinsky, A. N., Zhang, W., Chait, B. T. & Matunis, M. J. Proteomic analysis of the mammalian nuclear pore complex. *J Cell Biol* **158**, 915-927, doi:10.1083/jcb.200206106 (2002).

3 Gerlitz, G., Darhin, E., Giorgio, G., Franco, B. & Reiner, O. Novel functional features of the Lis-H domain: role in protein dimerization, half-life and cellular localization. *Cell Cycle* **4**, 1632-1640, doi:10.4161/cc.4.11.2151 (2005).

4 Garcia-Higuera, I. *et al.* Folding of proteins with WD-repeats: comparison of six members of the WD-repeat superfamily to the G protein beta subunit. *Biochemistry* **35**, 13985-13994, doi:10.1021/bi9612879 (1996).

5 Ulitzur, N., Harel, A., Feinstein, N. & Gruenbaum, Y. Lamin activity is essential for nuclear envelope assembly in a Drosophila embryo cell-free extract. *J Cell Biol* **119**, 17-25 (1992).

6 Gandre, S. & Kahana, C. Degradation of ornithine decarboxylase in Saccharomyces cerevisiae is ubiquitin independent. *Biochem Biophys Res Commun* **293**, 139-144, doi:10.1016/S0006-291X(02)00194-8 (2002).
